# From a Reductionist to a Holistic Approach in Preventive Nutrition to Define New and More Ethical Paradigms

**DOI:** 10.3390/healthcare3041054

**Published:** 2015-10-28

**Authors:** Anthony Fardet, Edmond Rock

**Affiliations:** Unit of Human Nutrition, Joint Research Unit 1019, Human Nutrition Research Center of Auvergne, French National Institute for Agricultural Research & Clermont University, F-63000 Clermont-Ferrand, France; E-Mail: edmond.rock@clermont.inra.fr

**Keywords:** preventive nutrition, healthy life years, sustainability, reductionism, holism, healthy core metabolism, intervention studies, food ranking, food structure

## Abstract

This concept paper intends to define four new paradigms for improving nutrition research. First, the consequences of applying a reductionist *versus* a holistic approach to nutrition science will be discussed. The need for a more focused preventive nutrition approach, as opposed to a curative one, will then be presented on the basis of the ‘healthy core metabolism’ concept. This will lead us to propose a new classification of food products based on processing for future epidemiological studies. As a result of applying the holistic approach, health food potential will be redefined based on both food structure and nutrient density. These new paradigms should help define a more ethical preventive nutrition for humans to improve public recommendations while preserving the environment.

## 1. Introduction

Although our production systems currently provide quality foods, they are far from being sustainable for various reasons. Healthier foods may have deleterious impacts on the environment and are often too expensive (*i.e.*, less accessible to the most disadvantaged socio-economic populations). Furthermore, widespread standardized diets do not always respect cultural habits or tradition (such as fast food), and rates of chronic diseases related to poor nutrition (e.g., obesity, diabetes, cardiovascular disease, certain cancers, osteoporosis, and sarcopenia) continue to increase dramatically worldwide [1,2].

To feed the world in a sustainable way by 2050, French experts have proposed that our daily consumption should not consist of more than 17% animal calories (and at least 83% plant calories) [3]. This is far from being the case in so-called developed and emerging countries, which, due to an increase in the middle classes, consume increasingly more animal products. For example, in France, we calculated that adults (18–79 years old) globally consume approximately 35%–40% animal products (in g/day) [4], with deleterious consequences for the environment, health and animal welfare.

The health and economic consequences are dramatic. For example, in France, although the theoretical life expectancy at birth has been increasing by three months every year (79 years for men and 85 years for women in 2013) [5], Healthy Life Years have decreased by approximately one year from 2008 to 2010 for women (from 64.6 to 63.5 years) [6]. Consequently, the number of years of unhealthy living is increasing each year with increasing human and financial burdens [7]. However, healthier diets and lifestyle would reduce risks of the main chronic diseases by at least 50%, reducing the number of years in poor health [8]. This knowledge means that a more balanced diet would enable healthy living probably largely beyond 70 years of age, which is a very achievable goal.

How did we get here? The first cause is the nutrition transition from a traditional diet rich in plant products and protective compounds to the so-called Western diet, which is rich in energy and animal products and poor in protective compounds [9]. While this transition has been slow in industrialized countries, it has occurred very rapidly in developing countries, where rates of obesity and diabetes are very high. There is also a lack of exercise due to an increasingly sedentary lifestyle. In addition to the nutrition transition, there is another more fundamental explanation for the development of poorly sustainable diets since the Industrial Revolution over a century ago: the dominant reductionist approach of our industrialized Western societies [10,11,12,13]. However, nutrition science is by nature a holistic discipline that considers all of its complex dimensions, including physiology, metabolism, food science, food sensory properties, physical exercise (energy output), socio-economic issues, behavior and lifestyle, cultural habits and environment, among others [14,15].

Based on these findings and understandings, we propose four new paradigms for a new preventive nutrition approach to increase Healthy Life Years [14,16,17].

## 2. Shifting from a Predominantly Reductionist to a Holistic Approach in Nutrition Research

### 2.1. Reductionism: A Typically Western Approach

In science, the application of the reductionist paradigm consists of splitting reality into separate entities and studying their functioning [14]. In medicine, this approach has saved millions of lives through the development of drugs and the discovery of the role of vitamins, e.g., where vitamin deficiencies are prevalent. In physiological sciences, reductionism has unraveled how the human body functions, be it digestively, metabolically, or genetically.

However, in nutrition sciences, notably food processing, this paradigm has led us to fractionate foods into their essential nutrients, *i.e.*, macro-, micro- and phyto-nutrients. The history of reductionism, or nutritionism, clearly parallels that of nutrition research and nutritional recommendations since the 19th century [13]. Scrinis divided the history of nutrition science and research into three periods: (1) the era of Quantifying Nutritionism; (2) the era of Good-and-Bad Nutritionism and (3) the era of Functional Nutritionism [13].

As Scrinis [13] wrote: “In the era of ‘quantifying nutritionism’, running from the mid-nineteenth to the mid-twentieth century, the focus of nutrition scientists was on discovering and quantifying the nutrients in foods and the nutritional requirements of bodies. The aim (…) was to identify the “protective” nutrients required for normal body functioning and growth, and particularly to prevent nutrient deficiency diseases. The era of “good-and-bad nutritionism” beginning in the 1960s was notable for the emergence of the novel idea of “good” and “bad” nutrients and for the emphasis on the need to avoid or reduce bad nutrients in particular. Negative dietary messages dominated this era, such as the low-fat campaign urging everyone to eat less fat. Dietary advice also shifted from the aim of preventing nutrient deficiencies to that of reducing the risk of chronic diseases, particularly heart disease. The present era of “functional nutritionism” began in the mid-1990s. Its distinctive feature is the rise of a more positive and targeted view of nutrients and foods as “functional” in relation to bodily health. Functional nutritionism also carries the expectation that particular nutrients, foods, and dietary patterns can enhance and optimize our state of health or particular bodily functions” [13].

More fundamentally, one finds the origin of reductionism in René Descartes (a French scholar of the sixteenth century) who viewed reality as the sum of components that could be divided into isolated entities, applying a mechanistic vision of the world. According to him, this decomposition and the resulting simplification would lead to the most appropriate explanation.

Before the reductionist paradigm, health was considered more holistically, as we have discussed in a previous paper [14]. Today, reductionism seems to have reached its limits as it has been pushed to its extremes in Western science [14]. Reductionism impacts on nutritional sciences are more deleterious than beneficial ones and include threats to biodiversity and the environment, a sharp increase in the prevalence of obesity and diabetes worldwide, the destruction of food health potential through fractionation/refining and ingredient recombination and deterioration of animal well-being through animal agriculture [14].

Why such a failure? Reality is complex and first obeys non-linear and multi-causal relationships, not linear cause-effect relationships as is often emphasized by reductionism. It is interesting that at the subatomic level, quantum mechanics tends to confirm this complexity of reality.

### 2.2. Holism: A Typically Oriental Approach

**Figure 1:**
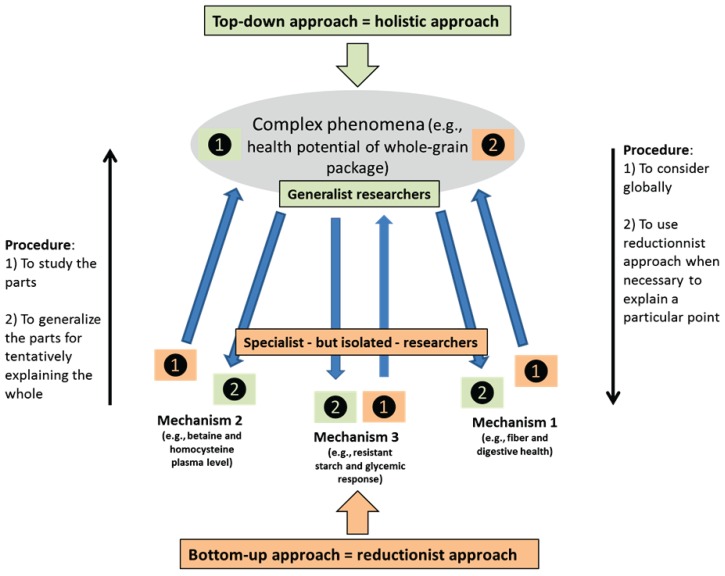
From reductionism to holism (from Fardet & Rock [14] with permission from the American Society for Nutrition^©^, Bethesda, MD, USA). The current conventional approach to research on food, nutrition and health, being reductionist, begins with isolated elements in food, as shown in the bottom half of this figure, and extracts data. By contrast, the holistic approach starts with foods in their whole form (or diet, lifestyle, or nutritional behavior), as shown in the top half of the figure, and thus begins with an idea that is then tested against information from aspects of the food.

Contrary to reductionism, holism is defined broadly by the view that a phenomenon is an indivisible whole and that the sum of its parts is not sufficient to define it. In other words, the whole is more than the sum of its parts, *i.e.*, 1 + 1 > 2 [14]. Holistic approaches are typical of oriental philosophies that tend to consider the entire system as superior to its parts. Thus, Oriental cultures tend to observe their surroundings from a more generalized perspective according to a top-down approach, from the general to the specific (Figure 1) [14]. For example, body and spirit are linked, as are the environment and human beings. As all is interconnected, abusing the environment and animals has consequences for human well-being.

The consequences of applying holism in nutrition sciences are numerous, from the preservation of biodiversity to the improvement of animal well-being through the protection of the environment [14]. Notably, a more holistic approach in food processing would lead technologists and/or food scientists to consider foods as systems that are not only a sum of their nutrients, but rather a package of bioactive compounds included in a complex food structure. A more holistic approach may also lead nutritional epidemiologists to associate disease risks with complex diets and/or overall quality of life rather than with only one nutrient, one food or food group.

### 2.3. Conclusion: Holism and Reductionism Are Complementary

This is not to say that reductionism is useless. It led to the discovery of nutrient functioning within human organisms, such as that of essential vitamins, and consequently to the prevention of nutritional deficiencies. As outlined previously [18], the most important issue is that while reductionism and holism are complementary, holistic approaches should be considered first before applying long and costly reductionist methods (Figure 1) [14]. Such an approach will avoid generalization from the specific according to a bottom-up approach. Nutrition sciences targeting disease prevention have to first be viewed as holistic disciplines, and when necessary, more reductionist approaches could be efficiently implemented.

## 3. Shifting from a Predominantly Curative Nutrition to a More Focused Preventive Nutrition

Reductionism has led us to apply a pharmacological approach to nutrition, viewing nutrients as drugs and nutrition as a means to correct already unbalanced diets rather than attempting to prevent chronic disease and to preserve health [11,14]. Consequently, most human intervention study designs are derived from pharmacological studies that use double-blind randomized controlled designs to compare the effect of a food or nutrient against a placebo. It is now evident that such a methodology cannot be applied to the effects of foods on health, as previously reported [11,19]. The development of functional foods, nutritional supplements and nutraceuticals clearly illustrates the tendency to see nutrients as drugs and nutrition as pharmacology [20,21].

**Figure 2:**
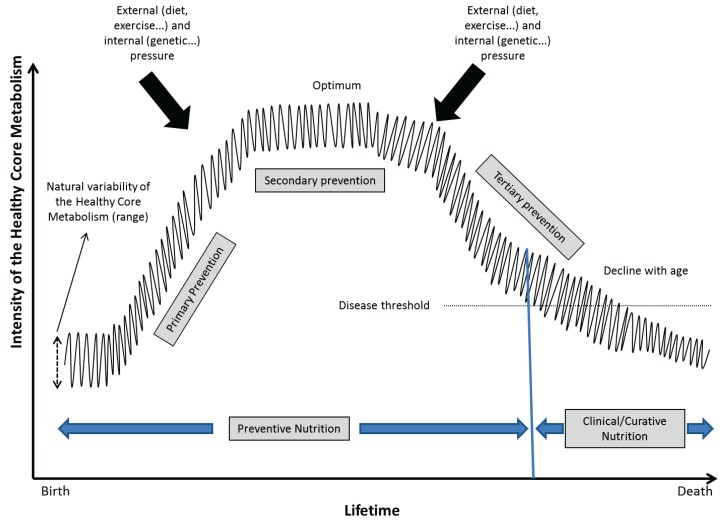
A schematic representation of the evolution of the Healthy Core Metabolism over a lifetime (from Fardet & Rock [16] with permission of Springer^©^, Berlin, Germany). The Healthy Core Metabolism is not constant at a defined time point but oscillates within a specific range depending on the genetic background of an individual. Oscillation may adapt to external stresses to help the whole organism regulate and maintain stability. The higher the optimal Healthy Core Metabolism, the longer it will take to decline upon ageing and the less it will be deregulated leading to a state of disease.

Moreover, the health care system and funding agencies are generally more interested in characterizing the unhealthy state, leading researchers to search for difference(s) between test and control groups. Rather, we should focus on the healthy state and characterize what is common to subjects after a nutritional intervention that is developed under what we have called the “Healthy Core Metabolism” concept (Figure 2) [16]. Indeed, a thorough and in-depth characterization of the healthy state is fundamental because it will facilitate the development of useful nutritional recommendations to preserve healthy conditions.

Adopting a healthy state approach would lead to new human intervention studies in “real life” conditions that follow healthy individuals in their natural environment and characterize their behaviors, physiology and food consumption in more realistic ways [16]. The advent of new technologies, such as smartphones with interactive applications, can be useful in following such behaviors in real time without substantial constraints [22].

“Finally, we hypothesized that true primary preventive nutrition should focus on the growth phase to reach the maximum capital of a given physiological function so that—Whatever the further decline—Healthy Life Years may approach or coincide with theoretical Life Expectancy” [16].

## 4. Ranking Foods in Epidemiological Studies According to Their Degree of Processing

A holistic approach views whole, natural and minimally processed foods as more beneficial to a person’s health than recombined and refined ultra-processed foods [17,20,21,22,23,24]. Consequently, processing should be developed to preserve the integrity of foods rather than to destroy it [25,26,27]. In contrast to ultra-processed foods, functional foods, supplements and nutraceuticals, whole natural and minimally processed foods are characterized by natural nutrient interactions necessary to act synergistically within an organism [10,20,21,28,29].

Therefore, the third paradigm we propose is to consider the extent of food processing in food ranking for epidemiological studies. In food frequency questionnaires and food pyramids, foods are generally clustered according to their botanical original or animal species, *i.e.*, fruits, vegetables, grains, dairy, or red and white meat. The problem is that the most conclusive, solid and relevant scientific evidence has been obtained by considering foods according to their degree of processing [29]. Indeed, the populations most affected by the increasing prevalence of diet-related chronic diseases are those who consume the most ultra-processed foods. By contrast, populations affected the least by chronic diseases and those having the highest life expectancy are those consuming the most natural, whole and minimally processed foods [30,31,32,33].

Within each typical food group, the degree of processing may vary considerably, from minimally processed to ultra-processed: for example, an apple may be eaten whole, canned with syrup, as a purée or as refined juice, and each degree of apple processing corresponds to a different health potential. We can provide such examples for each food group.

This is not to say that ultra-processed foods are useless, but rather that they should be used with parsimony as niche products, e.g., when practicing intense physical exercise, rapidly satisfying a little hunger, or not having time to eat fresh and/or natural complex foods, or even for soldiers or astronauts. Today, ultra-processed products tend to become predominant, such as in very large cities or in the poorest populations, leading to dramatic increases in the prevalence of chronic disease and subsequent expensive health care costs.

In addition, this new classification of food should be taught from primary school and may encourage the agro-food industry to deliver more healthy foods. Education is therefore essential in helping citizens make better food choices and in obliging food companies to adapt to these new demands.

## 5. Redefining Food Health Potential

As we have previously discussed, reductionism has led to the fractionation and refining of natural whole foods to isolate ingredients for further recombination, with an important loss of micro- and phyto-nutrients and thus a lower health potential. Reductionism (or “nutritionism”) has also led us to consider foods as only a sum of its nutrients. This quantitative view of food is only a part of the whole picture. Recent data clearly emphasizes the important role of food structure in its health potential, what is called “the matrix effect” [17,34,35].

**Figure 3:**
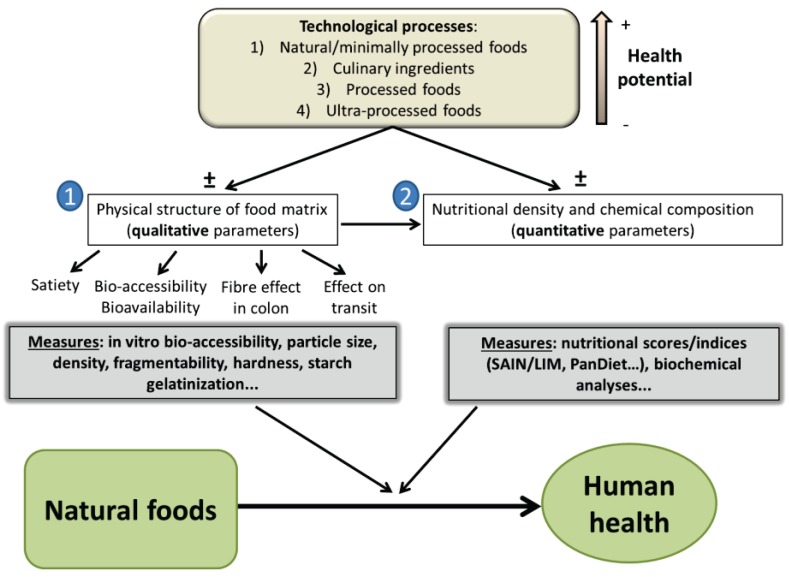
A new paradigm for the relationship between food processing, food health potential and human health, with an emphasis on food transformation (from Fardet *et al.* [34]. with permission from the American Society for Nutrition^©^, Bethesda, MD, USA). We propose that food health potential should be first defined by both the food’s structure and nutrient density [39] and that the impact of processing on these factors should be more systematically and extensively measured. We also propose that processing conditions either decrease or improve food health potential [25]. SAIN/LIM score: the SAIN score evaluates nutrient density by calculating the unweighted arithmetic mean of the percentage adequacy of the food positive nutrients; the LIM score calculates the mean content of disqualifying nutrients in 100 g of food. PanDiet score: A measure of the Probability of Adequate Nutrient Intake that uses the National French and US Dietary Surveys.

It is very clear that food health potential depends on both nutrient composition and food structure properties (Figure 3), such as cohesiveness, nutrient interactions and hardness [36]. In other words, two distinct foods with similar nutrient compositions but different matrices may have very different health potentials in humans as demonstrated by pasta and bread made of similar *durum* wheat [37]. In this study, bread and pasta elicited significantly different glycemic responses, which may be important for managing glucose metabolism in diabetic subjects. In the end, the most important physiological effect of food structure properties is its influence on nutrient bioavailability, and subsequently the synergy of their action within the human organism [38].

## 6. Conclusions

We think that the application of these four new paradigms in nutritional sciences, *i.e.*, a holistic approach, studying the healthy state, new food classifications and considering food structure, will allow us to completely reconsider health food potential and nutritional recommendations. For example, the new Brazilian dietary guidelines released at the end of 2014 are the first to propose holistic nutritional recommendations based on food pyramids that are built according to the degree of food processing [40].

Although this paper focused on nutrition, to be even more holistic, it is worth remembering from the standpoint of health that not only is it a person’s diet but also his or her lifestyle in general that contributes to overall health, including social environment, smoking and physical exercise.

In addition to reductionism, holism is now indispensable if we want to reconcile mankind with its environment (including both animals and nature), health and diets. This will involve an important shift in our way of thinking and to completely reconsider our way of leading studies in nutritional sciences. This is a very motivating challenge in the effort to develop sustainable diets by 2050.
